# Coronavirus Disease 2019-Related Acute Ischemic Stroke: A Case Report

**DOI:** 10.7759/cureus.10310

**Published:** 2020-09-08

**Authors:** Umar Farooque, Sohaib Shabih, Sundas Karimi, Ashok Kumar Lohano, Saurabh Kataria

**Affiliations:** 1 Neurology, Dow University of Health Sciences, Karachi, PAK; 2 Internal Medicine, Patel Hospital, Karachi, PAK; 3 General Surgery, Combined Military Hospital, Karachi, PAK; 4 Medicine, Peoples University of Medical and Health Sciences for Women, Nawabshah, PAK; 5 Neurology and Neurocritical Care, University of Missouri Health Care, Columbia, USA; 6 Neurology, West Virginia University, Morgantown, USA

**Keywords:** covid-19, ischemic stroke, absence of risk factors, humans, cerebrovascular accident

## Abstract

Coronavirus disease 2019 (COVID-19) is an active worldwide pandemic with diverse presentations and complications. Most patients present with constitutional and respiratory symptoms. Acute ischemic stroke remains a medical emergency even during the COVID-19 pandemic. Here we present a case of a patient with COVID-19 who presented with acute ischemic stroke in the absence of common risk factors for cerebrovascular accidents. A 70-year-old male patient, with no prior comorbidities, presented to the emergency department (ED) with fever, cough, and shortness of breath for four days, and altered level of consciousness and right-sided weakness with the sensory loss for one day. On examination, the patient had a score of 8/15 on the Glasgow coma scale (GCS). There was a right-sided sensory loss and weakness in both upper and lower limbs with a positive Babinski's sign. The pulmonary examination was remarkable for bilateral crepitation. On blood workup, there was leukocytosis and raised c-reactive protein (CRP). D-dimer, ferritin, thyroid-stimulating hormone (TSH), vitamin B12, and hypercoagulability workup were normal. Transthoracic echocardiography was also normal. COVID-19 polymerase chain reaction (PCR) detected the virus. Chest x-ray showed infiltrations in the left middle and both lower zones of the lungs in the peripheral distribution. Computed tomography (CT) scan of the chest showed peripheral and mid to basal predominant multilobar ground-glass opacities. CT scan of the head showed a large hypodense area, with a loss of gray and white matter differentiation, in the left middle cerebral artery territory. Magnetic resonance imaging (MRI) of the head showed abnormal signal intensity area in the left parietal region. It appeared isointense on T1 image and hyperintense on T2 image. It also showed diffusion restriction on the diffusion-weighted 1 (DW1) image with corresponding low signals on the apparent diffusion coefficient (ADC) map. These findings were consistent with left middle cerebral artery territory infarct due to COVID-19. The patient was intubated in the ED. He was deemed unfit for thrombolysis and started on aspirin, anti-coagulation, and other supportive measures. Patients with COVID-19 should be evaluated early for neurological signs. Timely workup and interventions should be performed in any patient suspected of having a stroke to reduce morbidity and mortality.

## Introduction

The severe acute respiratory distress syndrome coronavirus-2 (SARS-COV-2) appeared from Wuhan, China, at the end of 2019 and became a pandemic involving the whole world [1]. It can be asymptomatic or present with fever, fatigue, body aches, dry cough, dyspnea, and complications like acute respiratory distress syndrome (ARDS), severe pneumonia, acute kidney injury, myocarditis, multiorgan failure, and death [1,2]. Patients can also present with atypical gastrointestinal and neurological manifestations. Here we report a case of a patient with COVID-19 who presented with acute ischemic stroke without any predisposing conventional risk factors for cerebrovascular accident.

## Case presentation

A 70-year-old male presented to the emergency department (ED) with complaints of fever, cough, and shortness of breath for four days and altered level of consciousness, and right-sided weakness with sensory loss for one day. The patient was in his usual state of health when he developed fever and cough which was initially dry and later became productive with whitish sputum. The sputum was two teaspoons in quantity and difficult to expectorate. The patient also developed dyspnea during this time. The shortness of breath was sudden in onset, not associated with exertion, chest pain, sweating, palpitations, leg swelling, or any history of immobilization. The patient also denied any associated orthopnea or paroxysmal nocturnal dyspnea. Three days later, he developed an altered level of consciousness which was sudden in onset and associated with right-sided paralysis and loss of sensations. There was no associated neck stiffness, headache, or seizure. The patient did not have any comorbidities, including hypertension or diabetes. There was no family history of hypertension, or diabetes as well. He did not smoke or drink alcohol. There was no recent travel history or any prior history of similar complaints.

On physical examination, the patient was not oriented to time and place. His blood pressure was 130/80 mmHg, pulse was 72 beats/minute, respiratory rate was 26 breaths/minute, SpO2 was 82% on 100% non-rebreather mask, and temperature was 100 ^o^F. On neurological examination, the patient had a score of 8/15 on the Glasgow coma scale (GCS). Power was 1/5 in both upper and lower extremities on the right side and 4/5 on the left side in both lower and upper extremities. Babinski’s sign was also positive on the right side. Sensations were also absent in the right upper and lower limbs. There were no signs of neck rigidity. Lung examination was notable for harsh vesicular breathing with bilateral crepitation. Other systemic examinations were unremarkable.

On laboratory investigations, complete blood count revealed a total leukocyte count of 11,000 cells/mcL (normal range: 3,400 to 9,600 cells/mcL), hemoglobin of 10.7 g/dL (normal range - 13.2 to 16.6 g/dL) with a mean corpuscular volume (MCV) of 74 fL/cell (normal range - 80 to 100 fL/cell), and platelet count of 150,000/mcL (normal range: 135,000 to 317,000/mcL). Serum electrolytes including serum sodium, potassium, chloride, urea, and creatinine were within the normal range. Hemoglobin A1c was 5.4% (normal range: 4% to 5.6%). D-dimer, ferritin, thyroid-stimulating hormone (TSH), vitamin B12, and hypercoagulability workup were within normal limits. Transthoracic echocardiography was unremarkable. Real-time polymerase chain reaction (RT-PCR) testing for SARS-COV-2 was performed using a nasopharyngeal swab which was positive. C- reactive protein (CRP) was elevated to 28 mg/L (normal value: less than 10 mg/L). Blood and urine culture did not yield any growth.

**Figure 1:**
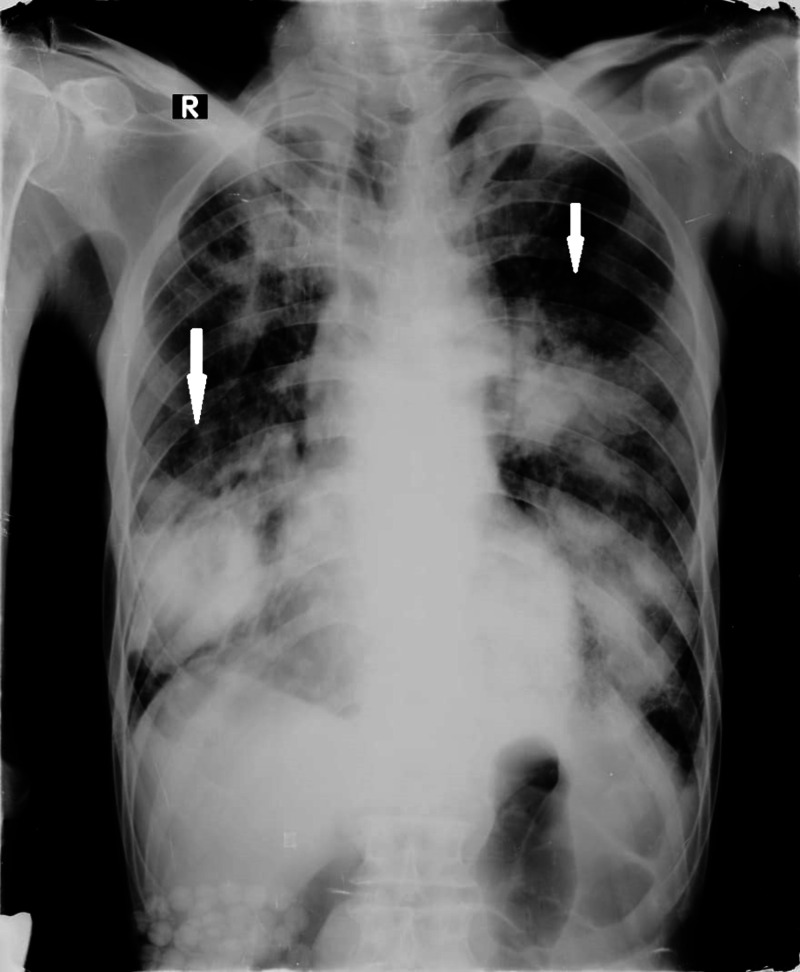
Chest x-ray This is a chest x-ray in the posteroanterior (PA) view showing bilateral infiltrates in both lower and left middle zones of lungs in the peripheral distribution

A chest x-ray showed bilateral airspace opacifications in both lungs, more pronounced in the left middle and both lower zones, with relative sparing of the left upper zone and peripheral distribution (Figure 1).

**Figure 2:**
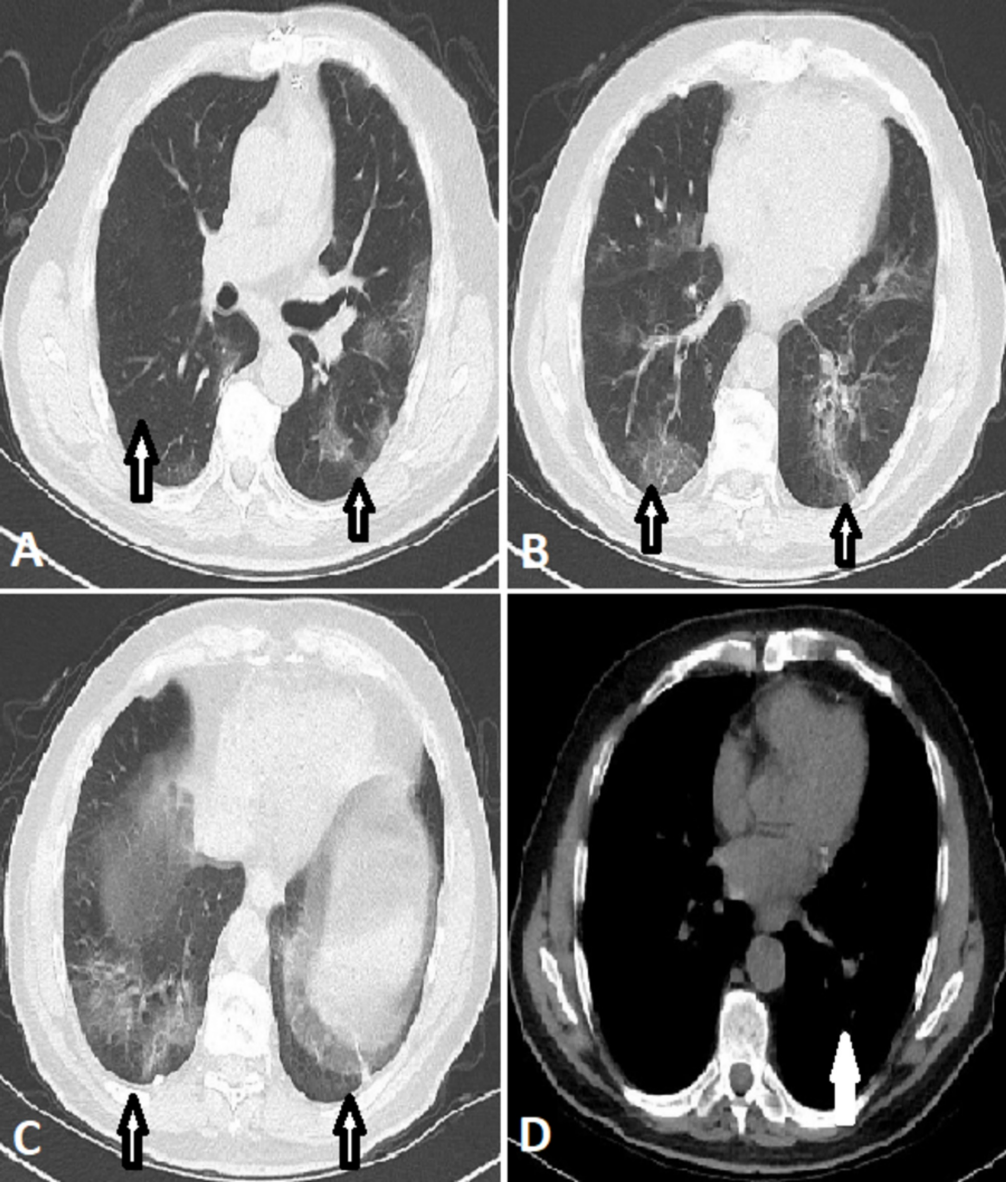
CT scan of chest These are axial views of CT scan of the chest showing multiple ground-glass opacities in the left middle and both lower zones in the peripheral distribution

CT scan of the chest showed multilobar ground-glass opacities with peripheral and mid to basal predominance. There was air space consolidation in the left lower lobe. There was no significant mediastinal lymphadenopathy. These findings were consistent with COVID-19 (Figure 2).

**Figure 3:**
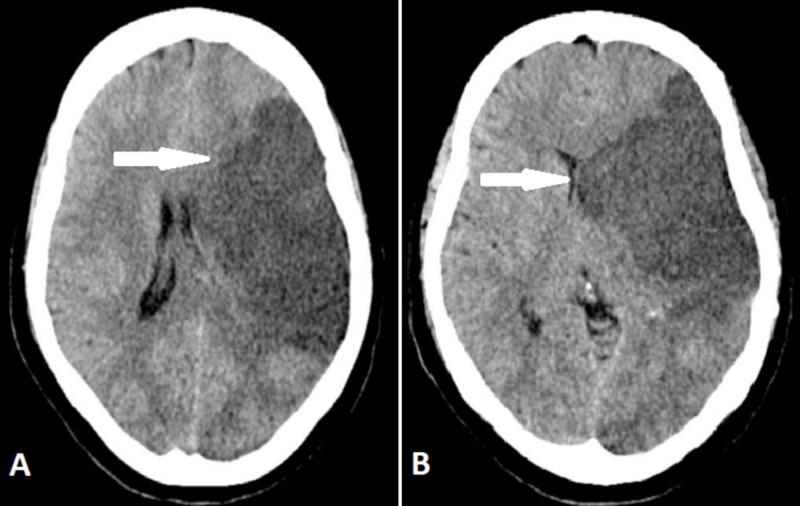
CT scan of head These are CT scans of the head without contrast in axial view showing hypodense area in the left middle cerebral artery territory

CT scan of the head showed a large hypodense area in the left middle cerebral artery territory with a loss of gray and white matter differentiation and effacement of the ipsilateral left lateral ventricle. These findings were consistent with left middle cerebral artery territory infarct (Figure 3).

**Figure 4:**
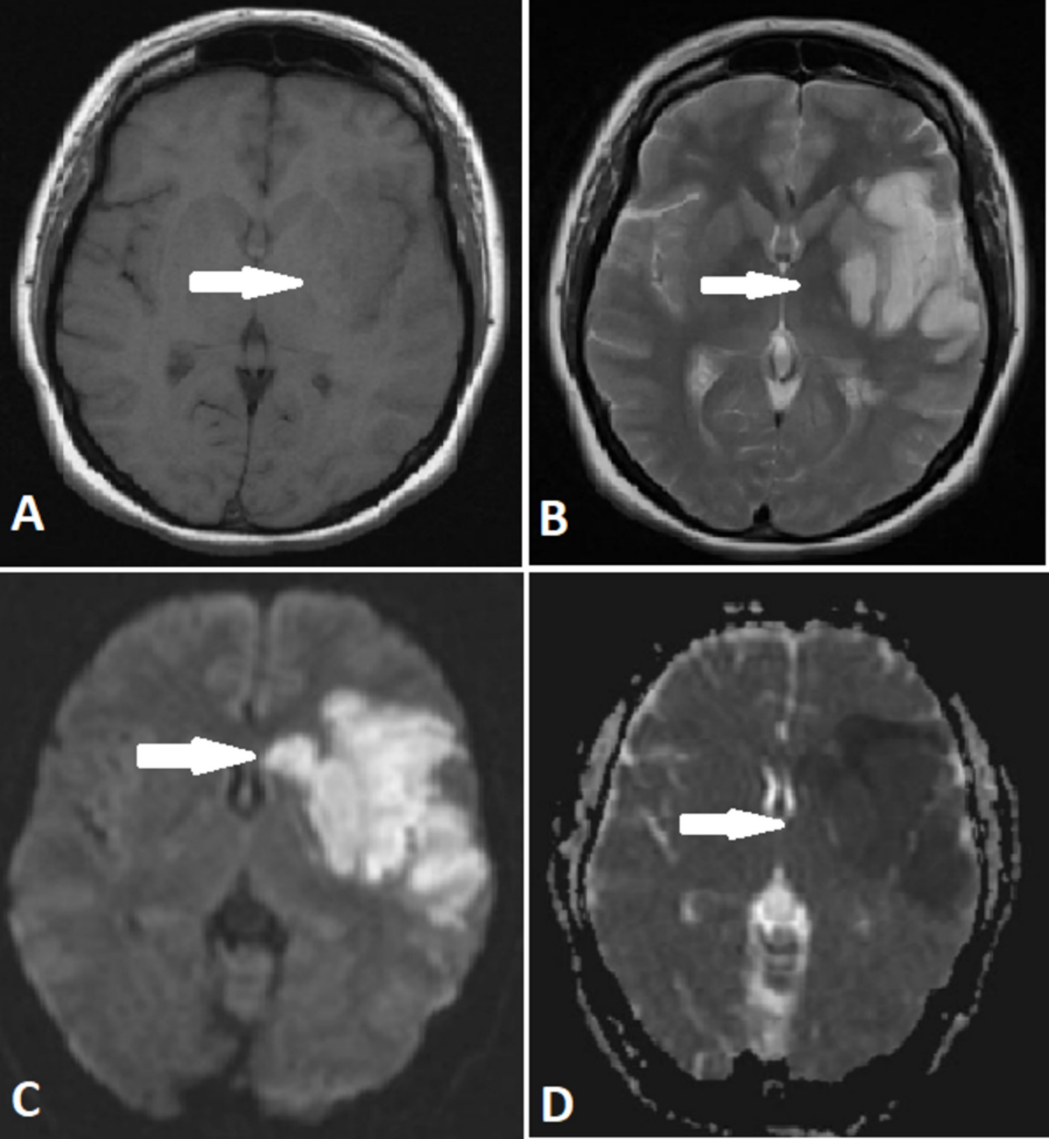
MRI scan of head in axial view (A) T1 scan showing isointense signal in the left parietal region. (B) T2 scan showing abnormal high signals in the left parietal region. (C) Diffusion-weighted 1 (DW1) scan showing a large area of restricted diffusion in the left parietal region. (D) Apparent diffusion coefficient (ADC) scan showing low signals in a large area of the left parietal region

Magnetic resonance imaging (MRI) of the head showed abnormal signal intensity area in the left parietal region, which appeared isointense on T1 image, and hyperintense on T2 image and showed diffusion restriction on diffusion-weighted 1 (DW1) image with corresponding low signals on apparent diffusion coefficient (ADC) map. These findings were consistent with left middle cerebral artery territory infarct (Figure 4).

The patient was intubated in the ED for hypoxemic respiratory failure. He was not deemed a suitable candidate for thrombolysis or neuro-intervention. So consequently, the patient was shifted to the intensive care unit (ICU) and started on aspirin 150 mg twice daily, subcutaneous low molecular weight heparin (LMWH) 0.6 ml twice daily, and intravenous dexamethasone 1 cc twice daily. The family eventually decided to pursue comfort measures.

## Discussion

COVID-19 is a current worldwide pandemic with diverse complications. Recent literature has shown multiple neurological manifestations including cerebrovascular accidents in patients with severe infection [3,4].

A single-center study of 221 patients showed that cerebrovascular events were more common in patients with stroke risk factors such as hypertension, diabetes, and previous history of cerebrovascular accidents. The mean age of patients who developed stroke was also higher and the stroke group had a higher frequency of hepatic and renal dysfunction [5]. SARS-COV-2 infection has also been shown as an independent risk factor for ischemic stroke [6]. Here in our case also, the patient developed acute ischemic stroke in the absence of conventional vascular risk factors.

The exact pathophysiology behind these cerebrovascular accidents is yet to be established. Infectious/inflammatory syndromes are associated with an increased risk of stroke, probably due to different mechanisms involving prothrombotic state, alterations in lipid metabolism, platelet aggregation, and modifications in endothelial function [7]. SARS-COV-2 binds to the angiotensin-converting enzyme receptor. This binding results in a cytokine storm which leads to a hypercoagulable state in patients with COVID-19 [8]. Critically ill patients with SARS-COV-2 also show elevated d-dimer levels and platelet counts, which increases the propensity towards acute cerebrovascular episodes [9].

The current COVID-19 pandemic necessitates that extra measures be taken to provide care to stroke patients, along with measures aimed at minimizing the spread of infection. Paramedics should develop an infectious screening policy in all patients before bringing them to the hospital [10]. All suspected stroke patients should receive brain imaging within 20 minutes of arrival in the ED. Negative pressure carrier isolators can be used to isolate COVID-19 patients during neurovascular imaging. The possibility as to whether anticoagulant or antiplatelet agents may be superior in stroke patients with COVID-19 requires further consideration. Similarly, no data exists to suggest a clear-cut benefit or risk with using intravenous recombinant thromboplastin plasminogen activator (rt-PA) therapy. A low threshold for initiating intubation, mechanical ventilation, and general anesthesia may be required in patients with COVID-19 infection who are selected for mechanical thrombectomy to reduce exposure risk during the procedure [11].

The mortality rate in COVID-19 patients with stroke is very high [12]. Older age, high sequential organ failure assessment (SOFA) score, cardiovascular diseases, secondary infections, ARDS, acute renal injury, lymphopenia, and elevated liver enzymes, CRP, ferritin, fibrin, and d-dimers are some of the factors in COVID-19 cases which can identify patients at risk of in-hospital mortality [13].

## Conclusions

Patients of COVID-19 can present with cerebrovascular accidents. Stroke teams should be aware of this fact and screen the suspected patients for acute neurologic changes as soon as possible, so that management can be initiated in time, and morbidity and mortality can be reduced. In future, further analysis is needed on a larger scale to find out the true relationship between COVID-19 and ischemic stroke and its pathogenesis.
